# Commentary: Time perception deficits and its dose-dependent effect in methamphetamine dependents with short-term abstinence

**DOI:** 10.3389/fncel.2020.00263

**Published:** 2020-09-08

**Authors:** Shuohui Gao, Xiaoxiao Yao, Lihua Sun, Yang Lin, Xin Li, Wei Yang

**Affiliations:** ^1^Department of Gastrointestinal Colorectal Surgery, China-Japan Union Hospital of Jilin University, Changchun, China; ^2^Jilin Provincial Key Laboratory on Molecular and Chemical Genetic, The Second Hospital of Jilin University, Changchun, China

**Keywords:** addiction, methamphetamine, dopamine, behavior, abstinence

Methamphetamine, one of the most popular synthetic drugs, is a mental stimulant. According to the latest United Nations world drug report 2018, methamphetamine is the fourth-largest product of all illegal drugs, second only to cocaine and third among users of stimulants (World Drug Report, 2018). In China, by the end of 2018, there were more than 2,404,000 registered drug users nationwide, in which methamphetamine is already the “No. 1 drug.” Methamphetamine addiction brings many economic and social problems to the whole society. For individuals, drug addiction is generally considered to be a chronic, recurrent brain disease.

Methamphetamine dependents are often accompanied by various cognitive impairments. From simple reaction time tasks to memory, chronic methamphetamine use is associated with cognitive deficits for most domains, especially for impulsivity/reward processing and social cognition (Potvin et al., 2018). However, previous work seems to have overlooked a more basic factor, that is time perception. Time is one of the most important dimensions of the real world. Time perception is also considered to be the cornerstone of all kinds of cognitive processing (Block, 1990). The existing evidence of distortions in time perception in people with impulse control disorders (e.g., pathological gambling) and personality disorders (e.g., antisocial personality disorder) has revealed the accelerated sense of time, which could help us to better understand the nature of impulsivity and addiction (Wittmann and Paulus, 2008; Moreira et al., 2016; Paasche et al., 2018). Previous studies mainly focus on the impact of methamphetamine on time perception in rats. They report a classic finding, which is a methamphetamine-induced leftward shift, suggesting the accelerated internal clock speed and temporal overestimation (Williamson et al., 2008). The underlying mechanism of this effect mainly relates to the damage of methamphetamine to the dopaminergic system in the striatum and prefrontal cortex (Gouvêa et al., 2015; Petter et al., 2016; Soares et al., 2016). The loss of corticostriatal plasticity in the motor area of methamphetamine dependents also can be observed (Huang et al., 2017). In other words, the dopamine mechanism of time perception overlaps with methamphetamine's brain-damaged regions (e.g., prefrontal-striatum network, motor cortex) (Petter et al., 2016). The existing animal studies have preliminarily revealed the important role of the withdrawal period in time processing (Williamson et al., 2008). For humans, a lot of work has shown the partial recovery of dopamine transporter levels and cognitive function with abstinence (Volkow et al., 2001; Wang et al., 2004; Stock et al., 2019). However, there is still a lack of systematic investigation on the change of time perception of human methamphetamine dependents and the association with drug use history.

These understandings were addressed in a recent study published in the journal of Science Advances, Zhang et al. They recruited a large number of healthy people in communities and methamphetamine dependents at different periods of withdrawal in drug rehabilitation institutions. This study systematically explored the changes in motor timing and perceptual timing components of time perception in methamphetamine dependents, as well as their relationship with the amount of methamphetamine use before abstinence, using time reproduction and comparison tasks at different intervals. Experiment 1 investigated motor timing using a time reproduction task of 1~5 s. In this task, the classic Vierordt's law can be observed (Lejeune and Wearden, 2009). Specifically, short durations are judged as longer than they really are, whereas long durations are judged as shorter, with a non-difference time point in between. Zhang et al.'s results showed that the non-difference time point of methamphetamine dependents in the short withdrawal period (MS group) was 1 s earlier than that in the long withdrawal period (ML group) and healthy controls (HC). This was an advanced Vierordt's law, indicating that the internal clock speed of the MS group was accelerated. The methamphetamine groups had a lower coefficient of variation, an index of response variability, dividing the standard deviation of reproduced intervals by the mean value of reproduced intervals. The lower variability of behavioral responses in methamphetamine dependents suggested that they have a more concentrated time estimation tendency regardless of time scales and abstinent durations. Moreover, the behavioral indicators were significantly correlated with the amount of methamphetamine used before abstinence only when the MS group processed a longer time interval than 1 or 2 s. That is, this dose-dependent effect disappeared as abstinence progressed. On the one hand, the dosage of methamphetamine use can affect timing and time perception, which has been observed in rats (e.g., Cheng et al., 2007); on the other hand, the partial recovery may improve time perception. In experiment 2, perceptual timing was investigated by time discrimination tasks at short intervals (200~800 ms) and long intervals (1,400~2,600 ms). No inter-group differences were found in behavioral indicators (point of subjective equality and time sensitivity). There was only a significant negative correlation between the amount of methamphetamine use before abstinence and the point of subjective equivalent under the supra-second processing, indicating that the time interval associated with higher cognitive activities was likely to be overestimated in the early stage of withdrawal. Note that the behavioral inter-group differences only were observed in motor timing rather than perceptual timing in methamphetamine dependents. It takes into account the neural mechanism differences between these two kinds of time perception. Although there is a core network across all timing tasks in the brain, including the basal ganglia, cerebellum, dorsolateral prefrontal cortex, supplementary motor area (SMA), and right inferior parietal lobe (Macar et al., 2002), some regions (e.g., SMA) will show more active when performing motor timing compared with perceptual timing (Wiener et al., 2010). Zhang et al. (2019) found that the accelerated speed of motor timing rather than perceptual timing could persist at least 3 months after abstinence. To some extent, the authors highlight the important role of the motor cortex in methamphetamine rehabilitation. Future investigations with functional imaging are needed to elucidate this inference. Zhang et al. (2019) recruited methamphetamine dependents to provide new evidence to the time perception deficits. Although some human studies in this field have been done (e.g., Wittmann et al., 2007), Zhang et al. had a big sample size and incorporated more important factors that have not been examined before (e.g., abstinence, history of methamphetamine use). Moreover, pure drug use is also beneficial to clarify the true impact of methamphetamine on time perception. Commonly, humans perceive time based on an internal clock (Treisman, 1963; Gibbon, 1977). Dopamine agonists (e.g., methamphetamine) and antagonists (e.g., haloperidol) could increase and decrease the internal clock speed, respectively. (Williamson et al., 2008). In animal studies, methamphetamine leads to a horizontal shift to the left in the response curve. In contrast, haloperidol, which primarily antagonizes dopaminergic receptors, leads to a horizontal shift to the right in timing function. Zhang et al. (2019) observed a similar behavioral pattern in abstinent methamphetamine dependents and found that the effect of methamphetamine on time perception exists in human beings as well. More importantly, the dose-dependent effect occurs only when short-term abstinent subjects process supra-seconds. On the one hand, this point suggests that deficits in time perception may last at least for 2 months. On the other hand, the effect of methamphetamine on time perception also relates to the drug use before abstinence. More systematic results are shown here.

From the clinical perspective, the investigation of time perception in methamphetamine dependents can provide a key reference for future precise interventions. Zhang et al. (2019) mainly found that the alteration of motor timing, not perceptual timing, in methamphetamine dependents could persist at least 3 months after abstinence and the dose-dependent effects on time perception appeared in processing longer intervals. These detailed behavioral response patterns provide a reference for future precise interventions, in which researchers should pay more attention to short-term abstinent methamphetamine dependents with higher use dosage.

Recently, the behavioral performance in time tasks has been considered as treatment outcome. Young et al. (2018) explored the treatment response of motor timing in patients with substance use disorder (SUD), in which 74 alcohol and/or cocaine users were employed and needed to complete an 8-week treatment (group therapies, individual counseling, written work, and a psycho-educational lecture series). The behavioral set consisted of motor reaction task, spatial-tapping task, and Go-No-Go task, which could measure different aspects of motor timing (catching a target, reacting with a fixed interval, and making decisions when appropriate, respectively). The confidence score (self-efficacy) to abstain from alcohol and/or cocaine was self-reported as well. Participants would be tested at three points in time, including within 72 h of the start, after the treatment, and the 12-month follow-up. They mainly found that motor timing indexes predicted a 27% variance in alcohol self-efficacy change and a 25% variance in cocaine self-efficacy change after the treatment. This study highlights the prognostic value of motor timing in SUD. Following this idea, Zhang et al. (2019)'s results could be applied in the future diagnosis and treatment of methamphetamine dependents.

Some limitations of the original study should be noted here. First, female participants were not included in Zhang et al.. Although many human experiments on methamphetamine dependence have used only males, there is a robust gender difference in time perception (Block et al., 2000; Zhang et al., 2017). Specifically, women will overestimate temporal intervals. Second, the correlations between the dosage of methamphetamine use and behavioral indicators seem pretty weak. This makes people wonder if dopaminergic systems preferentially affect longer intervals over shorter ones. However, previous evidence has found that different timescales (from milliseconds to hours) are mediated by dopaminergic levels (Marinho et al., 2018). Therefore, future research should consider gender differences in time perception and dopaminergic systems and employ more participants to clarify these correlations. Some cognitive neuroscience techniques (e.g., electroencephalogram, functional magnetic resonance imaging) should also be applied to promote our understanding of neural mechanisms of Zhang et al.'s findings. These practices are beneficial for researchers to use non-invasive brain stimulation techniques (e.g., transcranial magnetic stimulation) to accurately treat the time perception abnormality of methamphetamine dependents. In summary, Zhang et al. (2019), based on a large human sample, revealed the time perception changes of methamphetamine dependents after withdrawal, laying the foundation for systematic brain function assessment, intervention strategies, and improvement of the quality of these patients in this field.

## Author Contributions

SG wrote the first draft. XY and LS made major revisions to the logic of this article. YL, XL, and WY participated in the discussion of the manuscript. WY provided critical revisions. All authors approved the final version of the manuscript for submission.

## Conflict of Interest

The authors declare that the research was conducted in the absence of any commercial or financial relationships that could be construed as a potential conflict of interest.
